# Determination of Lamb Wave Modes on Lithium-Ion Batteries Using Piezoelectric Transducers

**DOI:** 10.3390/s22134748

**Published:** 2022-06-23

**Authors:** Markus Koller, Gregor Glanz, Alexander Bergmann, Hartmut Popp

**Affiliations:** 1 Center for Low-Emission Transport, AIT Austrian Institute of Technology GmbH, Giefinggasse 2, 1210 Vienna, Austria; gregor.glanz@ait.ac.at (G.G.); hartmut.popp@gmx.at (H.P.); 2 Institute of Electrical Measurement and Sensor Systems, Graz University of Technology, Inffeldgasse 33/I, 8010 Graz, Austria; alexander.bergmann@tugraz.at

**Keywords:** lithium-ion batteries, Lamb waves, piezoelectric transducer, battery management system, structural health monitoring

## Abstract

This work presents a method to determine the type of Lamb mode (antisymmetric or symmetric) that propagates through a lithium-ion pouch cell. To determine the type of mode and the group velocity at a specific frequency, two- and three-transducer setups were created. For these setups, it is important that all transducers have the same polarization direction. Two transducers are affixed to the center of the cell at a distance of several centimeters from each other so that the group velocity can be determined. Using cross-correlation, the group velocity of the emerging mode can be calculated. The measurement setup and the processing method was first validated with experiments on acrylic glass and aluminum plates. The measurements were supported with FEM simulations and a numerically calculated model. The output voltages of the receiving piezo-elements obtained in the FEM simulation are in agreement with the underlying theories. The phase shift, which results from the output voltage of the piezo-elements mounted one above the other on different sides of the plate, shows the type of mode. The results of the experimental determination of the Lamb mode that propagates through a lithium-ion pouch cell were validated with a numerically calculated multi-layer model and therefore validate this novel experimental approach.

## 1. Introduction

Structural health monitoring (SHM) based on guided waves can be used in many applications to improve safety and reliability. SHM can help to reduce maintenance costs and therefore reduce the life-cycle cost of the structure [1]. To perform active SHM that directly measures the structural health, sensors must be installed on the structure. Piezoelectric transducers or optical fiber sensors are commonly used. Damage detection based on guided waves has already been performed in several publications such as [2,3,4]. In [2] it is shown how structural defects in composite aircraft structures can be detected using ultrasonic guided waves. Smithard et al. [4] embedded two piezoelectric transducers into a composite-honeycomb structure to detect impact damage. In this work, the authors compared a healthy structure with a damaged structure by measuring the amplitude and the waveform of a received ultrasonic wave.

Guided waves can also be used for the estimation of the state of charge (SOC) and state of health (SOH), or other state estimations (SOX), for lithium-ion secondary batteries (LIB). During charging and discharging, as well as over their lifetime, the mechanical properties of LIB change, which can be analyzed with the help of ultrasonic waves. The ions, which are transferred from anode to cathode and vice versa during cycling, cause the active material to swell and shrink with the SOC. Irreversible processes, such as forming of interfaces, lead to an increase of thickness over the lifetime [5]. Ladpli et al. [6,7] and Popp et al. [8] measured the time-of-flight (TOF) and the signal amplitude of the guided waves during cycling to estimate the SOC and the SOH. Popp et al. [8], for example, showed, with the help of two piezo transducers mounted on a 12 Ah pouch cell, that the TOF correlates with the SOC of the cell. They measured the TOF, which is the time between emitting and receiving the acoustic signal, with a simple comparator circuit as receiver and a MOSFET circuit as transmitter. Similar measurements were performed by Ladpli et al. [7]. To better understand the acoustic behavior of the LIB, the type of wave must be determined. However, the interpretation of such guided waves is still a challenging task due to the complexity of wave propagation and the underlying structure. Therefore, the aim of this work is to classify the wave packets that occur when piezoelectric transducers are excited on a LIB pouch cell. To classify these wave packets, measurements were taken and supported by simulations and numerical calculations. The numerical calculations include an FEM-based model and an acoustic multi-layer model of the LIB.

**Figure 1:**
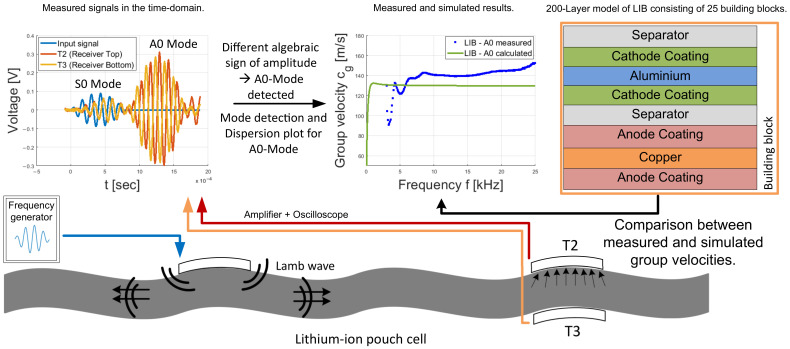
Graphical description of the used workflow to determine the different types of Lamb wave modes and for measuring parts of the dispersion plot of an LIB.

A LIB consists of several thin layers that are stacked together. The thickness of an LIB pouch cell is expected to be smaller than the wavelength of the generated longitudinal (P-) and shear (S-) waves. Therefore, in [7,8], it is assumed that Lamb waves, which belong to the class of guided waves, are formed. The reflections on the top and bottom of a LIB pouch cell lead to superimpositions that form wave packets (Lamb waves). These wave packets or guided wave modes can occur in symmetric or antisymmetric form. To check these assumptions, reference measurements were first made on aluminum and acrylic plates to distinguish between symmetrical and antisymmetric waves. Lamb waves show dispersive behavior, which means their propagation speed depends on the frequency and the plate thickness. By conducting the measurements, the dispersion behavior is visualized by generating a part of the dispersion curve, which is compared with the numerical calculations. The measurements were then repeated on four LIB cells. Figure 1 shows the overall setup and workflow to determine different Lamb waves on the LIB pouch cells.

Furthermore, with this workflow, the proposed simulation model shown in Section 5.3 can be used to determine the mechanical properties of an LIB. This can be done by comparing the measured dispersion plot, which shows the dispersive behavior of the Lamb wave, with the simulated dispersion plot. By knowing the dispersion plot of an LIB, a specific Lamb mode can be tracked.

There are various methods to measure the dispersion plot, for example, those shown in [9,10]. These measurement methods reconstruct the dispersion plot based on a B-scan image where the sensor position must be varied. Not all these measurement methods are suitable for LIBs and BMS applications. Therefore, this work shows methods that can be implemented in a BMS.

One of the main difficulties in estimating the TOF in LIBs is choosing an excitation signal that is suitable and capable of reproducing consistent results, especially when parameters such as the SOC and SOH of the battery are varied and the LIB changes its mechanical structure. Due to the limited size of LIB pouch cells, reflections on the edges of the propagating waves can occur. Together with different higher-order Lamb wave modes that exist at higher excitation frequencies, the measured signal at the receiving transducer can be a superimposition of multiple wave packets. This will cause varying amplitudes and wave packet lengths, especially when changing the parameters of the battery. Therefore, it will be very difficult to correlate the measured TOF with the SOC or SOH without deviations or outliers. To overcome these measurement problems, a three-transducer setup that allows the determination of the wave packet of a specific Lamb mode is described in this paper (see Section 5.1). By being able to identify the propagating zero-order modes together with the theoretical background of Lamb waves (see Section 2), conclusions about the material parameters that are dependent on the state of the LIB, such as the Young’s modulus of different layers, can be drawn. Based on this theoretical background, this paper presents an acoustic model that allows one to determine the propagation speed of the propagating A0 and S0 Lamb mode, and therefore makes it possible to define a suitable excitation signal for different types of lithium-ion pouch cells. Additionally, it can also be used to evaluate the change of the mechanical properties during cycling of the active materials and help to understand the underlying chemical processes of the LIB.

## 2. Theoretical Background

Guided wave problems are very well researched [1,11] and can be mainly categorized into Rayleigh waves (or surface waves), Lamb waves (waves in plates), and Stoneley waves. Since pouch cells have the shape of plates, it is assumed that mainly Lamb waves (plate waves) are formed. However, several publications exist where the wave speed in LIBs is described by calculating the longitudinal or pressure wave, which is not suitable for propagating Lamb waves and can lead to deviations between calculated and measured values. Because of this and to better understand the simulations conducted in this paper, the most essential parts of the Lamb wave theory are presented at this point.

Lamb waves travel through a solid body in two different modes, which are called symmetrical mode—S0—and antisymmetric mode—A0. In addition to these two most important so-called zero-order modes, an infinite number of higher order modes exist, which usually play a secondary role because they carry less energy and do not exist at all frequencies, such as the zero-order modes. There are already existing solutions for guided wave problems, for example, for Rayleigh, Lamb, and Stoneley waves. This work will concentrate on Lamb waves because of the layered structure of the LIB. As already mentioned, two type of modes exist: symmetric and antisymmetric modes.

**Figure 2:**
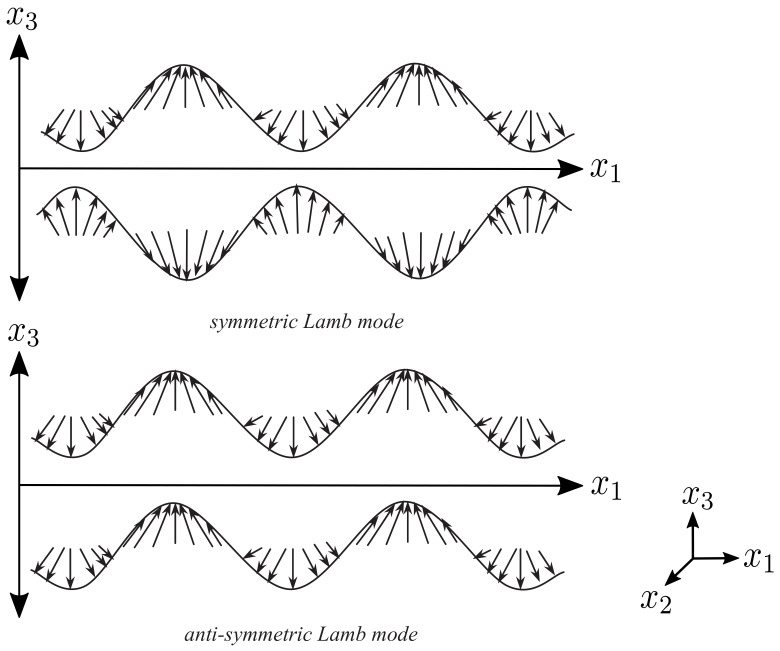
Displacement vector field (sum of in-plane and out-of-plane displacement) for A0 and S0 mode, modified with permission from [12].

Figure 2 shows an exaggerated representation of the displacement vector field on the surface of a plate [11] for the symmetric S0 and the antisymmetric A0 Lamb mode.

As shown in Figure 2, the displacement vectors for mode A0 point in the same direction along the top and bottom side of the plate. In comparison, the displacement vectors for mode S0 are directed in the opposite direction at any point on the respective surface. When placing a piezoelectric transducer on the top and on the bottom of the plate, the displacement on the surface of the plate causes a change in the strain of the piezo-element. This change in strain creates an equivalent voltage, which can be measured. The relationship between strain and sensor output voltage in a Cartesian coordinate system, where the indices one and two represent the surface direction (x,y) and the index three represents the direction in height (*z*), can be calculated using the following already simplified equation [13,14]:

(1) $$U=\frac{t_{p}d_{31}E_{p}}{\epsilon_{33}l_{p}^{2}\left(1-v_{p}\right)}\int_{l_{p}}\int_{l_{p}}\left(\varepsilon_{x_{1}}+\varepsilon_{x_{2}}\right)dx_{1}dx_{2}.$$

Since it is not possible to differentiate between the individual in-plane strains with a piezoelectric transducer, or by reducing it to a two dimensional problem, Equation (1) can be written in simplified form:

(2) $$U\approx \frac{t_{p}d_{31}E_{p}}{\epsilon_{33}\left(1-v_{p}\right)}\bar{\varepsilon }$$

where $E_{p}$ is the Young’s modulus of the piezoelectric material, $t_{p}$ the thickness of the transducer, $\epsilon_{33}$ the dielectric permittivity, $d_{31}$ a piezoelectric coefficient, $v_{p}$ the Poisson’s ratio, and $\bar{\varepsilon }$ the mean value of the applied strain. As can be seen in Equation (2), the sensor output voltage increases proportionally to the sensor thickness. Therefore, a thick piezoelectric transducer should be preferred for sensing applications. The displacement on the surface of the plate generated from Lamb waves causes a change in the strain of the mounted piezo-element. This change in strain should be equal when the transducers are mounted at the same position on both sides, as shown in the measurement setup Section 5.1. Therefore, the generated output voltage of both piezos must be equal when both piezos have the same polarization direction and a symmetric Lamb mode occurs. In contrast to this, the displacement of an antisymmetric Lamb mode produces an inverted output voltage on one of the two transducers. These theoretical considerations were used for the novel experimental setup, which will allow one to differentiate between two different Lamb wave modes. Another pair of parameters that can be used to determine changes in the mechanical properties of LIB are the phase $v_{p}$ and group $v_{g}$ velocities of the different modes. Since Lamb waves are dispersive, the speed of modes changes depending on the frequency *f*, from which the angular frequency *w* can be calculated. The phase velocity of the individual symmetric and antisymmetric modes can be calculated using the Rayleigh–Lamb equations as follows [11]:

(3) $$\frac{\tan(qh)}{q}+\frac{4k^{2}p\;\tan\left(ph\right)}{{\left(q^{2}-k^{2}\right)}^{2}}=0$$

(4) $$q\;\tan\left(qh\right)+\frac{{\left(q^{2}-k^{2}\right)}^{2}\tan\left(ph\right)}{4k^{2}p}=0$$

where *k* represents the wave number and *p* and *q* are defined as:

(5) $$p=\sqrt{\frac{w^{2}}{c_{l}^{2}}-k^{2}}$$

(6) $$q=\sqrt{\frac{w^{2}}{c_{s}^{2}}-k^{2}}$$

The phase velocities of longitudinal $c_{l}$, also called pressure waves and shear waves $c_{s}$, can be calculated using Equations (7) and (8).

(7) $$c_{l}^{2}=\frac{E\;(1-\nu )}{\rho \;(1+\nu )(1-2\nu )}$$

(8) $$c_{s}^{2}=\frac{E}{\rho \;2(1+\nu )}$$

By numerically solving Equations (3) and (4), the wave number *k* can be calculated over the frequency thickness product fd. It can be seen that Young’s modulus *E* and Poisson’s ratio ν, as well as the density ρ and the thickness d=2h, have an influence on the wave number and thus on the phase velocity of the LIB. For the following measurements, only the group velocity will be considered due to its simpler evaluation. The group velocity $c_{g}$ can be calculated based on the phase velocity by using Equation (9) [11].

(9) $$c_{g}=c_{p}^{2}{\left[c_{p}-\left(fd\right)\frac{dc_{p}}{d(fd)}\right]}^{-1}$$

where $c_{p}$ is the phase velocity.

## 3. Simulation

To prove the theoretical assumptions that the displacement and therefore the algebraic sign of the generated voltage from two opposite placed piezo-elements is inverted for the antisymmetric mode and the same for the symmetric mode, a two dimensional FEM simulation has been carried out. For the simulation, the program openCFS, version 20.9 [15] was used and the results were visualized with ParaView, version 5.8.1 by Sandia National Laboratories [16].

The simulation was conducted with acrylic glass because its material parameters, especially Young’s modulus *E*, are closer to those of an LIB than, for example, those of a metal such as aluminum.

Additionally, the computation time is significantly lower compared to aluminum because the propagation speed of the wave packets is smaller. Therefore, the discretization of the elements does not need to be as fine as for a metal, which saves computation resources. The chosen material parameters for acrylic glass can be found in Table 1, together with the parameters for aluminum, which will be also used for the analytic calculations performed in Section 5.2. Lead zirconate titanate (PZT) is used in the simulation for the piezoelectric transducer; the main parameters of this material are also given in the Table 1. For the simulation, other necessary parameters such as the elastic compliance constants and piezoelectric strain constants were taken from [17] and used to form the necessary tensors for a tetragonal crystal system from [18]. The model for the simulation consists of three transducers, one acting as transmitter and the other two as receivers. The receivers are located one above the other on different sides of the acrylic plate. With this setup, the measurements described in Section 5.2 are depicted.

For this transient simulation, a convergence study was performed where the element size of the mesh and the time stamp were varied. Multiple simulations were carried out by varying the time step between 0.01 μs and 0.16 μs and the element size between 0.1 mm and 1 mm. Comparing these simulations showed that the difference between the received signals using parameters smaller than 0.16 μs and 0.25 mm is insignificant for the purpose of this paper regarding the amplitude and the time-offset. Therefore, the above-mentioned values were chosen. The simulation results are also in accordance with the measured and calculated group velocities that are presented in Section 5.2.2, therefore proving its accuracy. A reasonable calculation time was also achieved with these values. To show that the algebraic sign of the voltage generated from the two receivers is the same for the symmetric mode and different for the antisymmetric mode, these two modes should not interfere with each other. By choosing a certain frequency and distance between the sending element and the two receiving elements, the difference in the propagation speed between these two modes will allow this desired separation. The chosen frequency in the conducted simulation was 50 kHz and the distance between the transducers was 200 mm. In general, the propagation speed of the symmetric S0 mode is higher than that of the antisymmetric A0 mode, which means that this mode will reach the two receiving piezo-elements first. Because of the faster propagation speed, the S0 mode will also reach the boundaries of the plate first, where it will be reflected, and will travel back through the plate until it reaches the opposite side of the plate, be reflected again, and so on. Therefore, it is possible that the reflected S0 mode passes at the same time as the slower A0 mode at the receiving piezo-elements, which would lead to a superimposition of these two modes. In this two dimensional FEM-simulation, the attenuation at the boundaries and of the whole material is neglected, and for this reason the acrylic plate needs to have a certain length to prevent this above-mentioned superimposition. When creating the FEM-model, it is also important that the polarization of the receiving transducer, which is placed on the bottom of the acrylic plate, is rotated by 180 degrees compared to the transducer on the top of the plate, in order to represent the measurement setup. Additionally, the two piezo-elements that are placed opposite to each other might have some influence in the generated signals due to their mechanical properties. To avoid this, two separate simulations, one with the sender element and the top receiver element and one with the sender element and the bottom receiver element, have been carried out.

**Figure 3:**
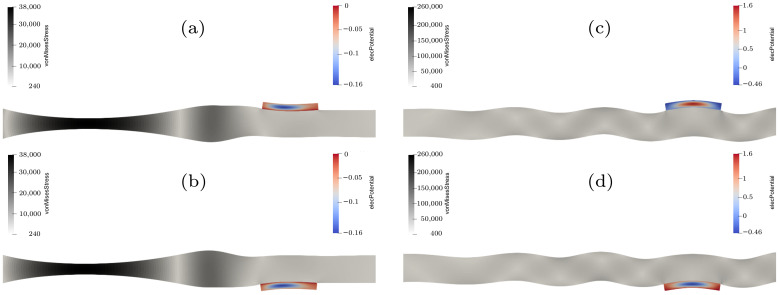
Results of the FEM-simulation of acrylic glass at 128.16 μs for the symmetric mode (**a**,**b**), where the displacement is amplified 80,000 times, and at 259.36 μs for the antisymmetric mode (**c**,**d**), where the displacement is amplified 10,000 times.

In Figure 3, the simulation results are visualized in the area of the two receiving piezo-elements. On the left side (Figure 3a,b), the displacement, which is increased by a factor of 80,000, 128.16 μs after the moment when voltage is applied to the sending transducer, can be seen. At this time, the S0 mode reaches the transducers, which can be verified by comparing the displacement that occurs on the top and the bottom of the plate, which face in alternating directions. The right side (Figure 3c,d) depicts the simulation after 259.36 μs, with a 10,000-fold increased displacement, where the antisymmetric mode reaches the receiving piezo-elements. The FEM-simulation confirms the results described in literature, where the top and bottom displacement of the plate, which is caused by the A0 mode, point in the same direction.

**Figure 4:**
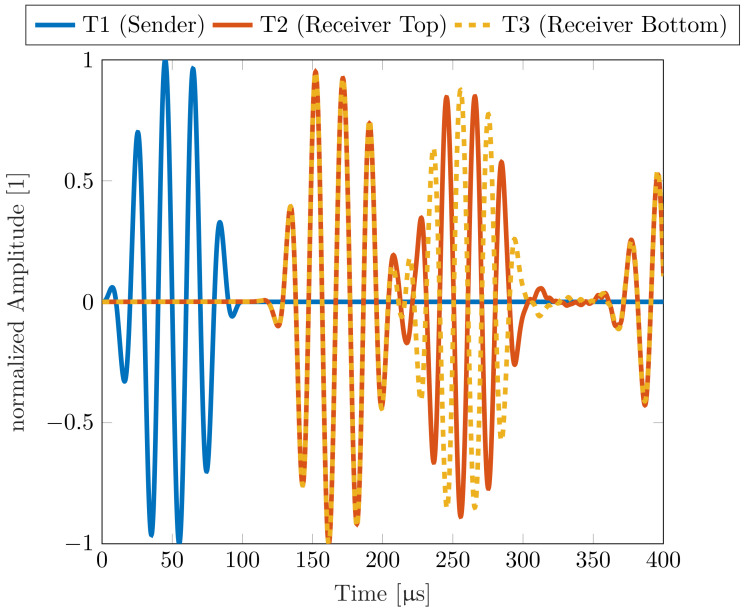
Simulated amplitudes of the piezo-elements at a distance of 200 mm at a frequency of 50 kHz for acrylic glass.

By looking at the generated voltage from the piezo-elements, which is shown in Figure 4, this phenomenon can be visualized even better. The S0 mode arrives after around 107 μs and has the same algebraic sign for both signals, while the A0 mode arrives at around 206 μs and has an opposite algebraic sign. Since the simulation was conducted without using damping coefficients and the piezo-elements are directly coupled with the acrylic plate, the order of the amplitude does not represent the values that can be found in literature or in measurements. This subject was not in the scope of the conducted simulation and therefore the values of the amplitudes in Figure 4 are visualized as normalized amplitudes.

The results of this FEM-simulation show that with this simple experimental setup, it is possible to distinguish between the two propagating zero-order modes by analyzing the received signal from the two receiving piezo-elements. The theoretical background that was proven with the FEM-simulation will be measured under real conditions for aluminum and acrylic glass. Afterwards, the previously used three transducer setup will be applied to an LIB and, for the first time, the propagating wave mode that travels through an LIB will be determined.

## 4. Signal Processing Method

This section shows how the group velocity was determined in the measurements. A common method to determine the similarities between two signals is the cross-correlation method. The output of the cross-correlation has the highest peak when the input signals are most similar. The cross-correlation for discrete time signals is defined as [1,19]:

(10) $$R_{xy}=\sum_{n=0}^{N-1}x\left[n\right]\;y\left[n-k\right]$$

Before the cross-correlation is applied, the envelope of the input and output signal is generated with the help of the Hilbert transform, where the Hilbert transform H{x(t)} for a continuous time signal is defined as [1]:

(11) $$H\left\{x\left(t\right)\right\}=\frac{1}{\pi }\int_{\infty }^{-\infty }\frac{x(\tau )}{t-\tau }d\tau$$

A discrete version of the Hilbert transformation can be realized using the fast Fourier transform (FFT) function as described in [11,20]. The Hilbert transformation can be used to form a complex signal z(t)=x(t)+jH{x(t)}. Calculating the absolute value from z(t) gives the envelope of the received and transmitted signal:

(12) $$e_{in}=\left|z_{in}\left(t\right)\right|$$

(13) $$e_{out}=\left|z_{out}\left(t\right)\right|$$

The discrete envelopes of both signals $e_{in}\left[n\right]$ and $e_{out}\left[n\right]$ are then cross-correlated:

(14) $$R_{e}=\sum_{n=0}^{N-1}e_{in}\left[n\right]\;e_{out}\left[n-k\right]$$

To find different modes, local and global maxima must be found in the cross-correlated signal.

(15) $$T_{\mathrm{peak},S0,A0,...}=findpeaks\left(R_{e}\right)$$

The group delay *T* can be calculated using the index of the detected maxima. With the help of the group delay, the group velocity can now be calculated using the distance *x* between the receiver and the transmitter:

(16) $$c_{g(S0,A0,...)}=\frac{x}{T_{\mathrm{peak},S0,A0,...}}$$

**Figure 5:**
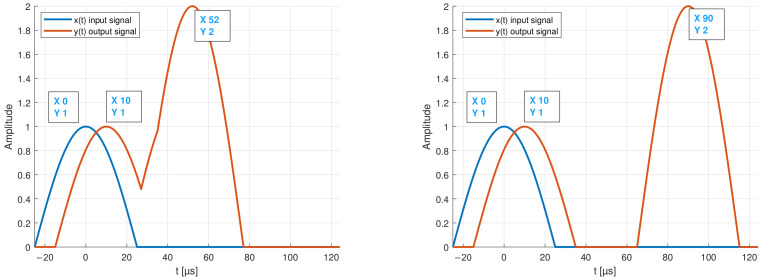
Input x(t) and output y(t) test signals. (**Left**): example of overlapping modes. (**Right**): envelope of two separate modes.

**Figure 6:**
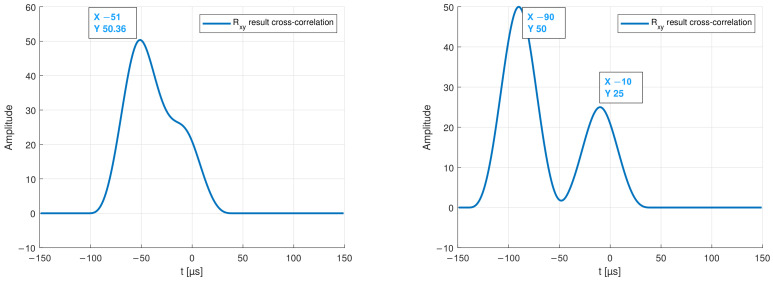
Output of the cross-correlation showing the maxima of two overlapping modes on the (**left**) graph, and of two separated modes on the (**right**).

Figure 5 and Figure 6 show the envelope of two example signals *x*(*t*) and *y*(*t*), as well as the result of the cross-correlation. The result shows that the output signals are most similar to the input signal at *t* = −90 μs and *t* = −10 μs.

Therefore, it can be assumed that two modes with different propagation speeds exists. However, if several signals, e.g., modes with different amplitudes overlay, a shift in the result of the cross-correlation can be detected. Figure 6 also shows that the signal with the smaller amplitude no longer generates a detectable peak in the result of the cross-correlation. Therefore, it is necessary to split the detected modes before. This can be a difficult task that, on the one hand, depends on the distance between the transmitter and the receiver piezo-element and, on the other hand, on the chosen excitation frequency.

**Figure 7:**
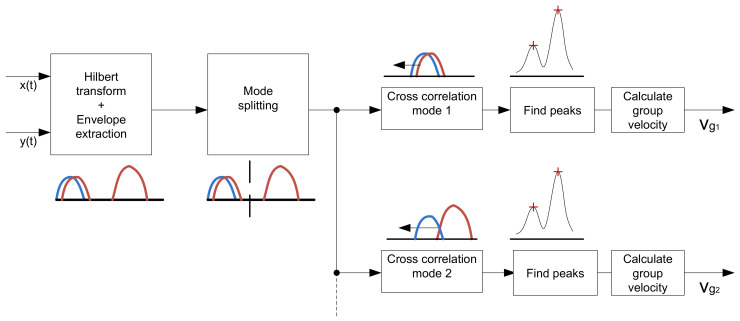
Block diagram of the mode detection algorithm.

However, if the distance between transmitter and receiver is sufficiently large and a proper frequency is chosen, modes can be found and separated using a peak detection algorithm. Figure 7 shows a block diagram of the algorithm used to determine the group velocity.

## 5. Experimental Method

This section shows how the type of Lamb mode and the group velocity can be determined with the help of a three transducer setup.

### 5.1. Measurement Setup

**Figure 8:**
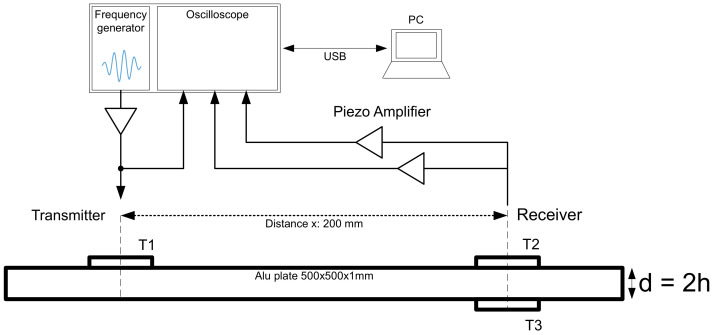
Three-transducer measurement setup.

A receiver amplifier circuit was developed for the further measurements. This amplifier increases the output voltage of the transducer by 52 dB at 10 kHz. Due to attenuation losses, the output voltage of the transducer is in the range of a few millivolts. To measure this output signal properly with an oscilloscope, a high amplification is necessary. To ensure such a high gain, two amplifier stages were implemented. The first amplifier stage is a non-inverting pre-amplifier that has a passive high-pass filter at the input. This high-pass filter has a cut off frequency of 2 kHz. The second amplifier stage consists of a differential amplifier. For this amplifier, only a single supply is necessary, because the ground potential at the input is raised by $V_{offset}$. The op-amp AD8092 from Analog Devices was used for the receiving amplifier. This dual op-amp has a gain-bandwidth product of 50 MHz and a slew rate of 145 V/μs. For the transmitter, an inverting power op-amp type MP111 from APEX Microtechnology was used. This power op-amp is necessary to deliver the required output current for the transducer at higher output frequencies. The USB Oscilloscope Analog Discovery 2 from Digilent, which also includes an arbitrary waveform generator, was used to automatically generate the excitation signal at specific frequencies. The excitation signal consists of four consecutive sine pulses, which are amplitude-modulated with a further sine wave. Such signals make it possible to excite the piezos with the desired frequency. If pulse-shaped square-wave signals were to be used as excitation signal, the resulting harmonics of the signal would lead the piezo to oscillate at a different frequency than desired. Figure 8 shows the measurement setup that is used for the group velocity measurements and the determination of the mode type.

**Figure 9:**
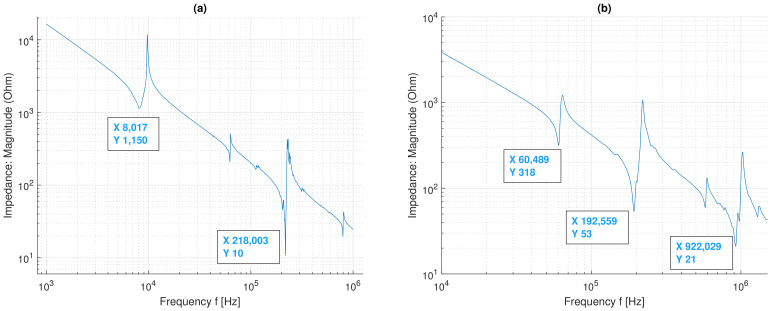
Impedance measurements (free air) showing the resonance frequency of the used disc transducer (**a**) and the block-shaped transducer (**b**).

Two types of piezo-elements were used in the measurements. Piezoelectric discs type AB1290B-LW100-R from PUI audio were used for the measurements on the acrylic plate and for the LIB. This transducer has a diameter of 12 mm and a capacitance of 8 nF at 1 kHz, according to the datasheet. For the measurements on the aluminum plate, block-shaped piezo-transducers with dimensions of 17 × 7 × 1 mm were used. In Figure 9a, the impedance measurement of the used piezoelectric disc is shown. It can be clearly seen that there are two main resonance frequencies at 8 kHz and 218 kHz. According to [17], for circular piezo-elements, these can be assigned to the radial and thickness extension modes. For the block-shaped piezo-elements, the resonance frequencies can be assigned to the length, width, and thickness of the transducer, as shown in Figure 9b.

With the help of the impedance measurement, the length and width resonance frequency ($f_{l}=60$ kHz, $f_{w}=192$ kHz) as well as the thickness resonance frequency ($f_{t}=922$ kHz) can be clearly assigned. Due to the limited bandwidth of the used transducers, only a limited frequency range where the excitability is high can be used [11]. At the resonance frequencies, the displacement of the transducers is high; therefore, the excitation should be in the range of these frequencies. Because of these excitability limitations and other physical limitations such as plate size and attenuation, only parts of the dispersion curve can be measured, which will be shown in the next section.

### 5.2. Reference Measurements

To verify the setup and the signal processing, reference measurements on three different plates were taken. Due to the well-known parameters of aluminum and acrylic glass, the dispersion plot can be calculated and compared with the measurement results. This is an important step before performing measurements on LIBs, which are more complex due to the high number of layers and different materials. The following measurement uncertainties can influence the calculation of the group delay and need to be considered:

- Group delay of the amplifier.
- DC offset due to noise.
- Superposition of different modes or reflections from the edges of the plate.
- Imprecise placement of the transducers.

#### 5.2.1. Aluminum Plate Measurements

The first reference measurements were done on a 1 mm thick aluminum plate with a size of 500 × 500 mm to prevent the influence of reflections. The transducers were placed in the center of the plate 200 mm apart from each other.

**Figure 10:**
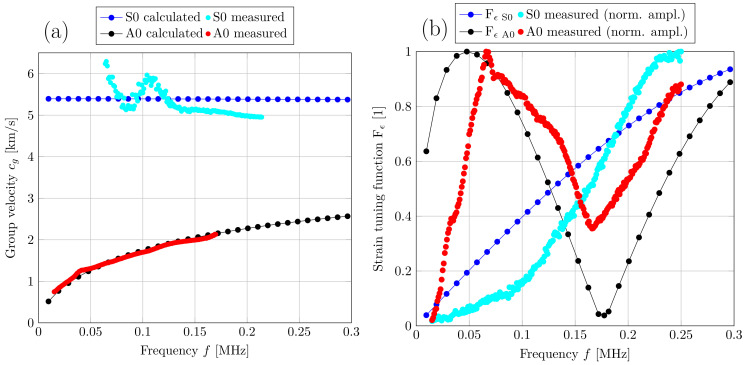
Group velocity measurements (**a**) and output voltage measurements (**b**) of the modes S0 and A0 for a 1 mm thick aluminum plate.

The group velocity can be calculated with the help of Equation (9). The dispersion plot in Figure 10a of an aluminum plate shows that only two modes (A0 and S0) occur at lower frequencies. The thicker the aluminum plate, the sooner other higher-order modes occur. With the algorithm shown in Figure 7, it was possible to reconstruct parts of the dispersion plot. At lower frequencies, the amplitude of the mode S0 is very low; therefore, it is difficult to detect and to evaluate it. The amplitude of the mode S0 starts to increase at higher frequencies. However, at higher frequencies, there is an offset in the S0 mode, which can be caused by the incorrect separation of the mode A0 or other reflections and noise. Furthermore, slight filtering or inexact compensation of the amplifier over the frequency range can cause deviations from the numerical calculated group velocities. Such measurement uncertainties have a larger influence on modes with higher group velocities, such as the mode S0. For example, 1 μs of delay which can be caused by an inaccurately compensated amplifier, will lead to a decrease of the measured S0 group velocity. For an excitation frequency of 150 kHz and a distance between the transmitter and the receiver of 200 mm, this means that the S0 group velocity decreases 142 ms^−1^. When measuring a mode with lower group velocities such as the mode A0, expecting the same setting as mentioned before, an additional group delay of 1 μs would only cause a decrease of 20 ms^−1^.

As shown in Equation (1), the output voltage of the transducer depends on the surface strain ε. Assuming an ideal bonding between the aluminum plate and the transducer, the strain response can be estimated for block-shaped or rectangular transducers by using the strain tuning function, which is derived in [1]. Equation (17) shows the strain tuning function,

(17) $$F_{\varepsilon }=sin\left(k_{A0}\;\frac{l_{p}}{2}\right)$$

where $k_{A0}$ is the wave number of the mode A0 and $l_{p}$ is the length of the transducer. Figure 10b shows a comparison between the strain response caused by the Lamb wave modes S0 and A0 and the measured output voltages of these modes. From Figure 10b, it can be seen that if the length of the transducer $l_{p}$ is an even multiple of $\frac{\lambda }{2}$, where $\lambda =\frac{c_{p}}{f}$ the surface strain and thus, indirectly, the output voltage is at its maximum. The minimum strain and therefore the minimum output voltage occurs when $l_{p}$ is an odd multiple of $\frac{\lambda }{2}$ [1].

**Figure 11:**
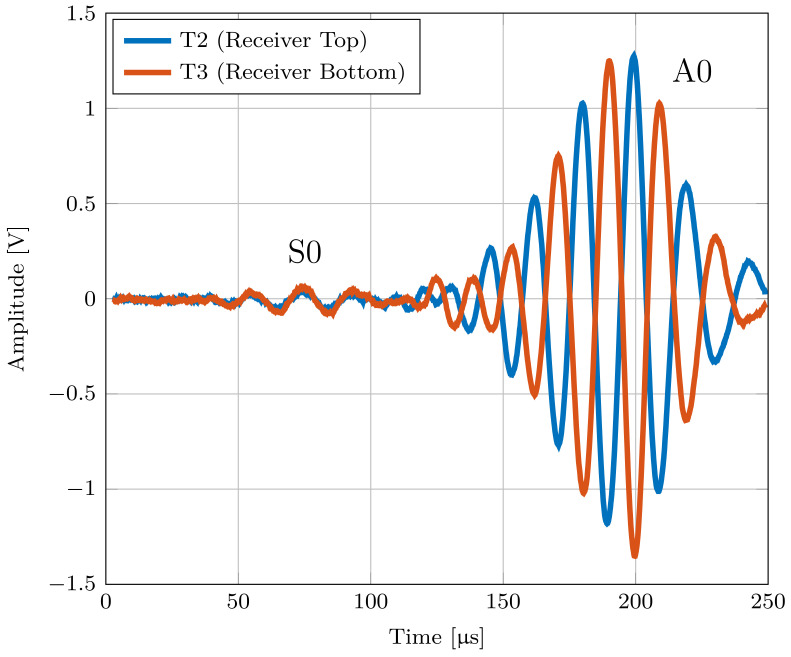
Output voltage of transducers T2 and T3, showing the behavior of the modes A0 and S0 for a 1 mm thick aluminum plate.

Based on the comparison between numerical and measured group velocities, it was possible to ensure that the measured mode is a symmetric or antisymmetric Lamb mode. With knowledge about the in-plane and out-of-plane displacement, which can be seen in Figure 2, the modes can now be assigned using the three-transducer setup. For this experiment, we compared the output voltage of transducers T2 and T3, which are placed on the opposite sides of the plate. Due to the same change in strain that both transducers experience during a symmetrical Lamb mode, the same output voltage is measured on both transducers. For comparison, an inverted output voltage is measured at transducer T2 during an antisymmetric Lamb mode, as shown in Figure 11.

Figure 11 shows the output voltages of T2 and T3 at an excitation frequency of 50 kHz.

#### 5.2.2. Acrylic Plate Measurements

**Figure 12:**
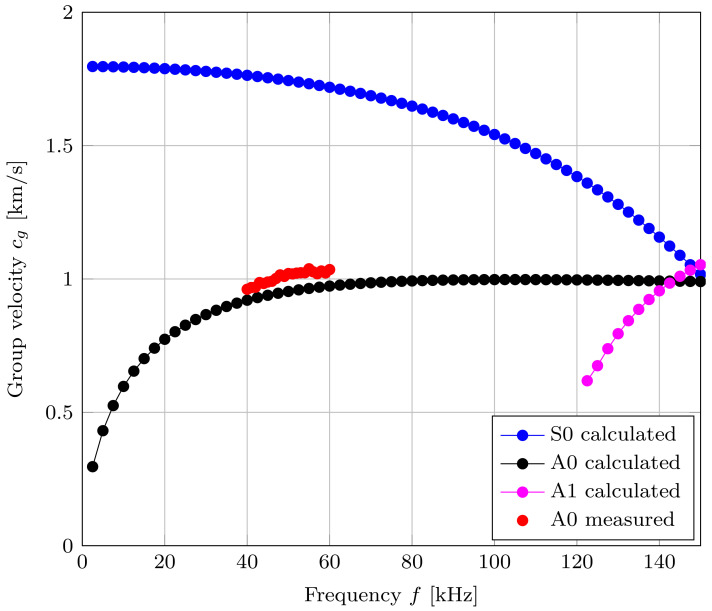
Group velocity measurements of the mode A0 for a 4 mm thick acrylic plate.

Another reference measurement was carried out on a 4 mm thick acrylic plate with a size of 500 × 500 mm. For this measurement, the distance between the receiver and transmitter had to be reduced to 100 mm due to the limited output voltage of the amplifier and the high attenuation of the material. Due to the shorter distance, modes A0 and S0 superimpose. The separation of mode S0 and therefore the determination of the group velocity was not possible at this distance. Because of the superposition of both modes, the cross-correlation result leads to an increase in the measured group delay compared to the analytically calculated values. Figure 12 shows the comparison of the measured and numerically calculated group velocities for an acrylic plate. Due to the thickness of the plate, a higher-order mode (A1) occurs at a frequency of 122 kHz, as shown in Figure 12. The high attenuation of the acrylic plate and the limited sensitivity of the transducer leads to a smaller measurement range, as can be seen in Figure 12.

### 5.3. LIB Measurements

**Figure 13:**
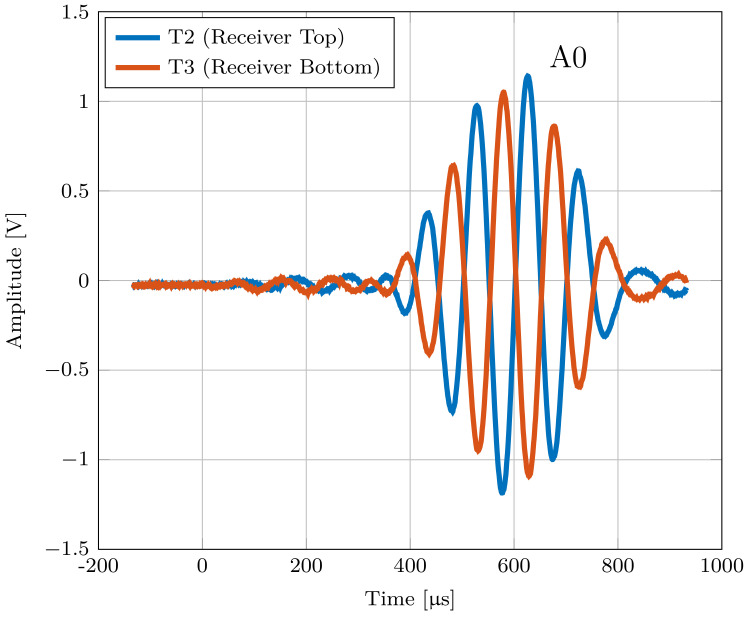
Output voltage of transducers T2 and T3 located on an LIB showing the behavior of the measured antisymmetric A0 mode at an excitation frequency of 10 kHz.

For the pouch cell measurements, four cells with lithium nickel manganese oxide (NMC) cathode material were used. Each cell has a capacity of 32 Ah and dimensions of 156 × 241 × 8 mm. The SOC of the cell was kept constant at ≈40% during all measurements. The measurements were done at room temperature. Due to the limited size of the battery, the distance between the transducers T1 and T2 had to be reduced to prevent superimposition coming from reflections from the boundaries. Therefore, the distance between the receiver and transmitter was varied between 4 and 8 cm. The used measurement setup is described in Section 5.1. To validate whether the measured waves are Lamb waves, the output voltages of the transducers T2 and T3 were measured first. With these output voltages, it is possible to distinguish between the symmetric and antisymmetric modes, as shown previously. To ensure that only the zero-order modes S0 and A0 occur, the excitation frequency is kept very low. Figure 13 shows the output voltages measured at T2 and T3 at 10 kHz and it can be seen that the measured mode that propagates through a Lithium-ion pouch cell can be classified as an antisymmetric Lamb mode A0.

At an excitation frequency of 10 kHz and a distance between T1 and T2 of 4 cm, the resulting group velocity is $c_{g}$ = 100 ms^−1^. To measure the dispersion plot of the classified mode A0, the transducers T1 and T2 were attached to the center of four different cells from the same type with a distance between them varying from 4 to 8 cm. The transducers were attached by using superglue. The transmitter was excited with four subsequent modulated sine pulses with a frequency between 3 and 25 kHz. As in the previous measurements, the algorithm shown in Figure 7 was used to determine the group velocity.

**Figure 14:**
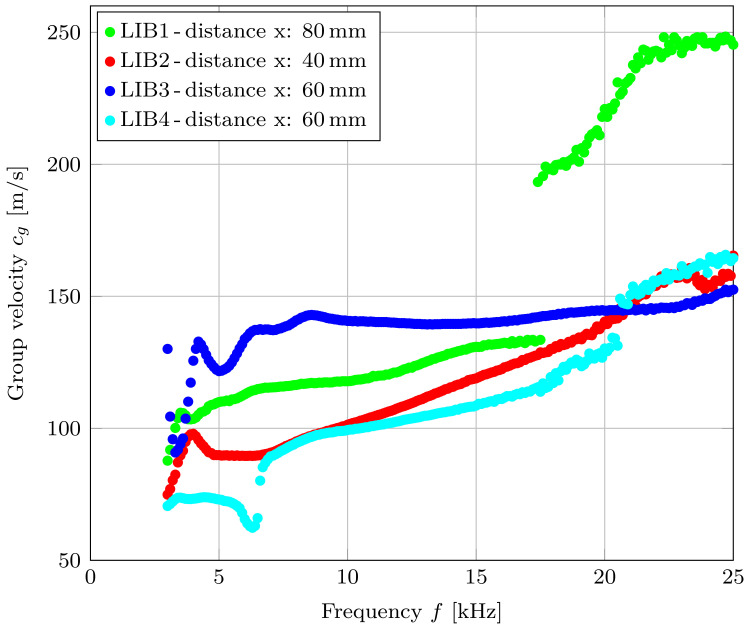
Group velocity measurements of the classified A0 mode on four different LIBs.

Figure 14 shows the measurement results of the group velocities, which were carried out on four LIBs (1–4) of the same type. The measured group velocities vary between ≈60 ms^−1^ and ≈170 ms^−1^, with exception of the outliers in the LIB1 measurements. These outliers could be an indication of higher-order modes, which are already appearing at a frequency of 17.4 kHz. Due to the higher distance between receiver and transmitter (8 cm), higher order modes are already starting to form wave packets. These waves packets could interfere with the measured A0 mode. It can be assumed that higher order modes can also be measured on the other cells (LIB2–LIB4) if the distance between the receiver and the transmitter piezo is further increased. Even though the distance between the emitting and receiving piezo-elements of LIB3 and LIB4 are the same, the group velocity is different over most of the measured frequency range. One possible explanation could be that the two cells did not have the exact same SOC and some small deviations regarding this value occurred. Furthermore, the manual gluing of the transducers could also influence the measurement results. Due to the different thickness of the applied adhesive, the coupling between the transducer and the LIB can be affected. The differences in the measured propagation velocities of different LIBs of the same type will be part of the authors’ future research. Additionally, it is also interesting how the group velocity of the antisymmetric Lamb wave behaves during cycling and whether this behavior varies between two different cells of the same type.

To estimate the group velocity of the cell, a simplified multi-layer model of the used pouch cell was created. The numerical solutions were determined using the software The Dispersion Calculator [21], version 1.11.2 by Armin Huber. The calculation of dispersion curves for multi-layered media is very computation-intensive; therefore, the dispersion curve was only calculated up to a frequency of 15 kHz. In addition, the calculation of higher-order modes was omitted, since this work mainly deals with the determination of the mode A0 and S0. These measures can reduce the computing time significantly.

Table 2 shows the model parameters used for each layer within the pouch cell. Most of the parameters used were obtained from the literature. The individual layer thicknesses were taken from previous measurements of cells with a similar capacity and thickness.

**Figure 15:**
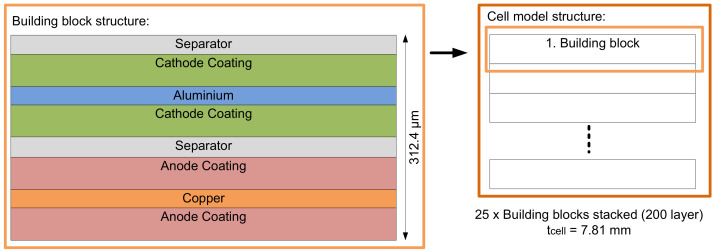
Multi-layer model of the pouch cell.

To simplify the structure of the cell model, it was created using several building blocks, as shown in Figure 15. One building block consists of an anode, cathode, and separator. These building blocks, each with a thickness of 312.4 µm, were stacked until the cell had a total thickness of 7.81 mm. As a result, a model with a total number of 200 layers (25 building blocks) was created. The thickness of 7.81 mm corresponds approximately to the thickness of the cells used in the measurements.

The calculations showed that the main influencing factor of the group velocity is the Young’s modulus *E* of the individual layers. In particular, the low Young’s Modulus of the separator and the coating reduces the group velocity of the mode A0 and S0 drastically.

**Figure 16:**
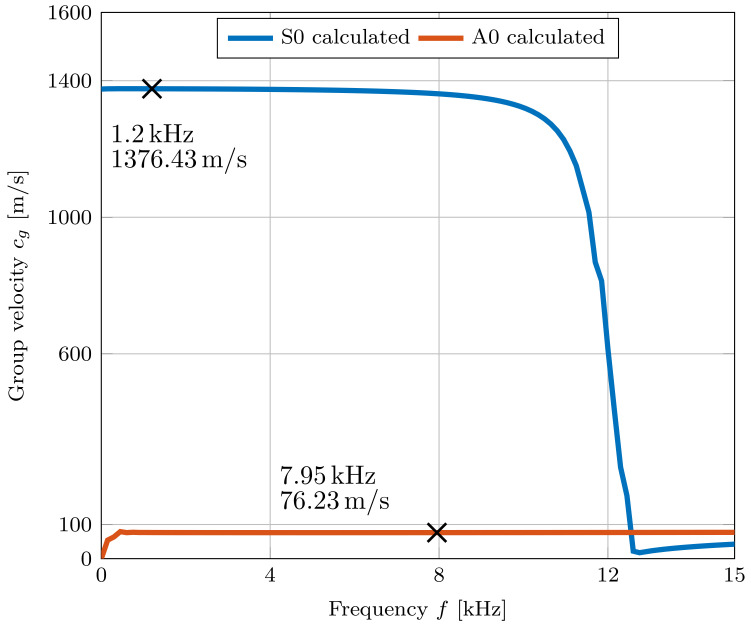
Resulting dispersion curve of the pouch cell multi-layer model with 200 layers and a total thickness of 7.81 mm. For two specific frequencies the resulting group velocities for S0 and A0 are shown.

Figure 16 shows the resulting dispersion curve of the multi-layer model. It can be seen that the group velocity of the mode A0 is 76 ms^−1^ at 7.95 kHz.

The group velocity of mode S0 starts to decrease drastically at 10 kHz and starts to approach the mode A0 at higher frequencies. S0 achieves a maximum group velocity of 1376 ms^−1^. Due to the total thickness of 7.81 mm and the material parameters of the different layers, the mode S0 is already overlapping with the mode A0 at 12.6 kHz.

The model confirms that due to the high number of layers and the resulting thickness, the group velocity of the mode S0 is already similar to the velocity of the mode A0 at quite low frequencies between 12.6 kHz to 17.4 kHz. This means that an overlapping of the received signals between these two modes takes place in this frequency range and, which is therefore not an optimal excitation range for determining the group velocity of the A0 mode.

The group velocity of the mode A0 was in a similar range in the measurements (≈60 ms^−1^− 170 ms^−1^) as well as in the model (≈76 ms^−1^). The parameters shown in Table 2 only serve as a first approximation and can differ from the parameters of the used LIB. Due to possible deviations in the parameters, the group velocity of the model can differ from the measured ones. However, this model can serve as a first approximation and can be further improved with more accurate data from the layers of the actual used LIB.

## 6. Conclusions

This work shows how to distinguish between the symmetric S0 mode and the antisymmetric A0 mode using a three-transducer setup. First, the experimental setup consisting of three piezo-elements with the same polarization direction were mounted on an aluminum and acrylic plate, as shown in Figure 8. The Lamb waves propagating through the plates could be assigned to their corresponding mode by identifying the phase shift and the algebraic sign of the two output voltages generated by the receiving transducers. The performed FEM simulation confirmed the underlying theory and the measurement results. Furthermore, the group velocity of the wave packets was measured over a certain frequency range using an algorithm based on cross-correlation. For these measurements, it must be ensured that there is sufficient distance between the transducers and the edges to avoid superimpositions. By calculating the group velocities numerically, the assigned modes of the measured results could be validated for the above-mentioned materials. In addition, the maximum amplitude of the respective mode for an aluminum plate was measured and compared with the result of the strain tuning function. The measured amplitude showed good agreement with the calculated strain tuning function.

Subsequently, the established experimental setup was used on an LIB pouch cell. Parts of the dispersion curve for four LIBs with three different distances between the transducers were measured. Because of the phase shift and the alternating algebraic sign of the generated signals, the propagating wave could be identified as an antisymmetric A0 mode. To validate these results, a multi-layer model representing the LIB was created. To simplify the structure, the model was divided into building blocks, where each block had the specific material parameters of the different components used in an LIB. The blocks were stacked until the cell thickness of 7.81 mm was reached. With the help of this multi-layer model, it was possible to show that the calculated group velocity of the A0 mode is in a similar range as the previously measured values. Therefore, it could be proven that with this novel experimental setup, not only the group velocity, but also the mode type that propagates through an LIB pouch cell could be determined. As a result, a certain area of the dispersion diagram for an LIB could be established for the first time.

To achieve an even better accordance between the numerical calculations and the measurements, the parameters used for the multi-layer model must be further adapted to the mechanical parameters of the used cell. Additionally, the exact number of layers and the real thickness of each layer could, for example, be provided by a CT image of the cell. Using the proposed measurement setup and the simulation model, the requirements to build a universal method for estimating the different states of an LIB were fulfilled. With this new experimental setup, it is possible to determine the A0 mode and thus to find the excitation signal free of superimposition and higher order modes. This drastically increases the reproducibility of the estimations based on mode tracking. By combining the information resulting from a more precise version of the numerical model, the measurements of the group velocities, and the amplitude of the occurring mode, conclusions could be drawn about the mechanical properties, for example, the Young’s modulus and the change of thickness of the cell. Furthermore, through the observation of the mode occurring in an LIB, conclusions can be drawn about the SOC and SOH, which the authors wish to examine in future research.

## Figures and Tables

**Table 1:** Material parameters and dimensions for acrylic glass, the piezo-elements, and aluminum used for the simulation and the numerical solution.

	Acrylic Glass	Aluminum	Piezo-Elements PZT-5H
Density [kg/m${}^{3}$]	1180	2700	7500 [17]
Young’s modulus [N/mm${}^{2}$]	3000	70,000	-
Poisson ratio	0.42	0.35	-
Height [mm]	4	1	1
Length [mm]	600	-	8

**Table 2:** Mechanical properties and thickness of the different layers used in a LIB.

Layer	Thickness	Young’s Modulus *E*	Density ρ	Poisson’s Ratio ν
	[µm]	[GPa]	[kgm^−3^]	
Separator (PE/PP)	7.7	0.095 [22]	915	0.4
Cathode Coating	60	0.194 [22]	4700 [23,24]	0.01 [25]
Anode Coating	78	0.031 [22]	2200 [23,24]	0.01 [25]
Aluminum	13	70	2700	0.345
Copper	8	100	8900	0.3

## Data Availability

Data are available upon request from the corresponding author.

## Abbreviations

The following abbreviations are used in this manuscript:

BMS	Battery management system
CT	Computed tomography
FFT	Fast Fourier transformation
LIB	Lithium-ion secondary battery
NMC	Nickel mangan cobalt oxide
PE	Polyethylene
PP	Polypropylene
SOC	State of charge
SOH	State of health
SHM	Structural health monitoring
TOF	Time of flight
ε	Strain (1)
*E*	Young’s modulus (GPa)
$\epsilon_{33}$	Dielectric permittivity (Fm^−1^)
*v*	Poisson’s ratio (1)
*k*	Wave number (m^−1^)
ρ	Density (kgm^−3^)
λ	Wavelength (m)
fd	Frequency thickness product (Hz·m)
$c_{p}$	Phase velocity (ms^−1^)
$c_{g}$	Group velocity (ms^−1^)
$c_{s}$	Shear wave velocity (ms^−1^)
$c_{l}$	Pressure wave velocity (ms^−1^)
